# Continuous venovenous hemodiafiltration using cytokine-adsorbing hemofilters as adjuvant therapy for anaerobic descending necrotizing mediastinitis: a case report

**DOI:** 10.1186/s13256-019-2123-7

**Published:** 2019-07-05

**Authors:** Dijana Popevski, Magdelena Popovska-Cvetkova, Katerina Ignevska, Rodney A. Rosalia, Zan Mitrev

**Affiliations:** Zan Mitrev Clinic, Skopje, Republic of Macedonia

**Keywords:** Descending necrotizing mediastinitis (DNM), Anaerobic septic shock, Extracorporeal blood oxygenation

## Abstract

**Background:**

We describe a combinatorial intensive care approach and discuss the critical factors that allowed us to successfully manage a life-threatening case of acute anaerobic septic shock triggered by descending necrotizing mediastinitis.

**Case presentation:**

We admitted a 38-year-old critically ill Kosovar Albanian man to our intensive care unit because of clinical manifestations of severe sepsis. His condition had worsened in the previous 2 weeks following unsuccessful antibiotic therapy for tonsillitis complicated by retropharyngeal abscesses. Computed tomography and intraoperative observations identified abscesses in the anterior and middle mediastinum regions and the distal part of the neck, directly on the border with the left lobe of the thyroid gland. Cultures indicated infections with α-hemolytic *Streptococcus* and *Clostridium* species: High procalcitonin and lactate levels, blood gas analysis, poor peripheral capillary oxygen saturation, and severe hemodynamic instability pointed to a case of acute septic shock. The entire treatment consisted of an aggressive antibiotic regimen, transthoracic and mediastinal surgical evacuation of the abscess, vacuum sealing drainage with a pleural chest tube, continuous venovenous hemodiafiltration using cytokine-adsorbing hemofilters, and extracorporeal blood hyperoxygenation.

**Conclusions:**

Efficient treatment of severe anaerobic sepsis resulting from descending necrotizing mediastinitis should build on a multidisciplinary approach. In support of first-line therapies with targeted antibiotics and surgical debridement, clinicians should consider alternative therapies such as continuous venovenous hemodiafiltration with cytokine-adsorbing hemofilters and hyperoxygenation.

**Electronic supplementary material:**

The online version of this article (https://doi.org/10.1186/s13256-019-2123-7) contains supplementary material, which is available to authorized users.

## Introduction

Descending necrotizing mediastinitis (DNM) is an acute, life-threatening disease caused by infections originating in the head and neck area. Immediate antibiotherapy, control of the source of infection, and surgical debridement of the affected tissue are the cornerstones of treatment [1]. The localization of the infection directly influences the mortality rate [2], and failure to control the microbial metastasis can lead to hemodynamic impairment, multiorgan failure, and septic shock [3–5].

A negative factor in the treatment of DNM-induced septic shock is the presence of polymicrobial anaerobic strains [6], particularly in those cases including clostridial species [7, 8]. Prognosis is poor once anaerobic septic shock is established [9, 10], as a consequence of the complex underlying disease, the source of infection [11], and the evolution of anaerobes toward antibiotic resistance [12].

This case report details our early initiation strategy of continuous renal replacement therapy (CRRT) combined with extracorporeal blood purification and hyperoxygenation in the management of sepsis. Furthermore, our work highlights the importance of multidisciplinary intensive care in the successful management of a 38-year-old man with acute anaerobic septic shock due to DNM.

## Case presentation

We admitted a 38-year-old Kosovar Albanian man, an entrepreneur, to our emergency department in a critical state. He has never smoked and has no prior medical history or family history of illnesses. He reported enjoying an occasional alcoholic drink.

His symptoms had started after physical exercise 2 weeks before. He was then diagnosed at a local clinic with tonsillitis (lacunar angina) complicated with several retropharyngeal abscesses. He was prescribed first-line oral antibiotics; the treatment failed, and his clinical condition drastically worsened in the following days, which prompted the referral to our hospital.

On clinical examination, he was tachycardic with a heart rate of 130 beats/min and blood pressure of 100/70 mmHg, heavily somnolent with a Glasgow Coma Scale score of 10, and hypoxemic and cyanotic with a ratio of partial pressure of arterial oxygen to fraction of inspired oxygen (PaO_2_/FiO_2_) of 66 mmHg and a mean arterial pressure of 82 mmHg. His peripheral capillary oxygen saturation was 75% while wearing a 6-L O_2_ mask. We detected abnormal bronchial breath sounds and reduced airflow in the lower lobes.

**Fig. 1:**
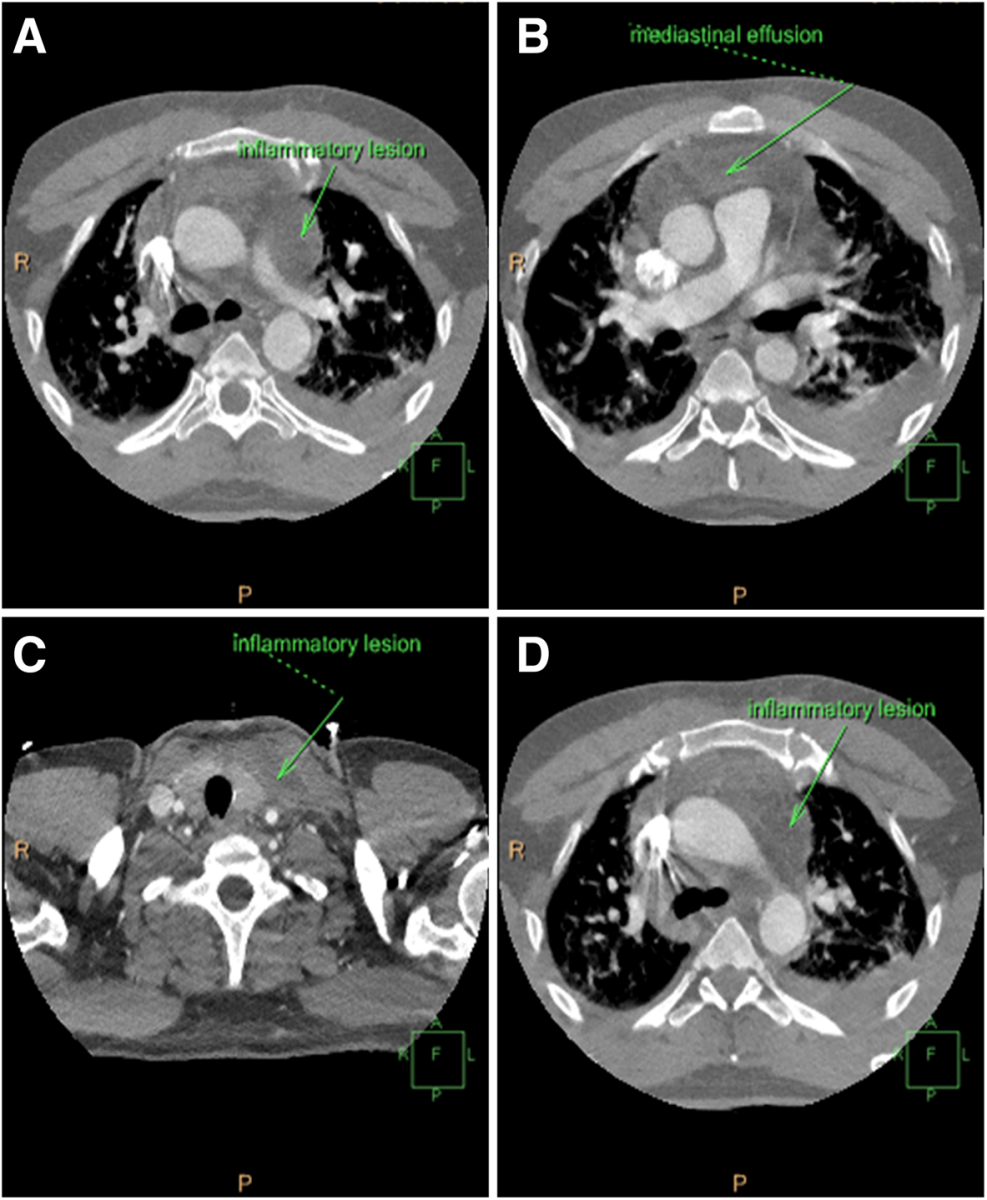
Preoperative computed tomographic (CT) scans of a critically ill patient displaying clinical signs of descending necrotizing mediastinitis. **a** CT scan showing increased density of the middle mediastinal space. Fluid collections in mediastinum and lymphadenopathy are shown (*arrow*). **b** CT scan showing increased density of the mediastinal space, fluid collections, and lymphadenopathy with bilateral pleural effusions (*arrow*). **c** CT scan showing fluid collections, as well as abscess formation left of the thyroid gland and ventral of the carotid arteries (*arrow*). **d** CT scan showing increased density of the middle mediastinal space, fluid collections in mediastinum, and lymphadenopathy (*arrow*)

Computed tomography (CT) indicated lymphadenopathy, inflammation, and fluid collection in the mediastinum accompanied by significant fibrin depositions (Fig. 1a, b). Abscesses were observed in the anterior and middle mediastinal regions (Fig. 1b, c) and the distal part of the neck, directly on the border with the left lobe of the thyroid gland (Fig. 1c, d). Other than weakness, the patient had no signs of any physical complications; moreover, neurological examinations did not reveal major abnormalities. The patient had slightly delayed motor responses and a normal pupil size and reaction to light, but his speech was incomprehensible.

The patient was admitted to the intensive care unit (ICU), where he was sedated (propofol, induction dose 2.0 mg/kg, maintenance dose 0.07–0.21 mg/kg/hr), intubated, and placed on mechanical ventilation with bilevel positive airway pressure FiO_2_ of 70%. We administered intravenous aminophylline 250 mg every 12 hr. As a gastroprotective therapy, we used ranitidine 50 mg every 8 hr. The continuous fluid therapy consisted of 200 ml/hr NaCl 0.9% and 50 ml/hr glucose 5% for the first 24 hr. We initiated parenteral nutrition using Aminoven 10% (40 ml/hr; Fresenius Kabi, Graz, Austria) and a fat emulsion (20 ml/hr; INTRALIPID® 20%; Fresenius Kabi).

**Fig. 2:**
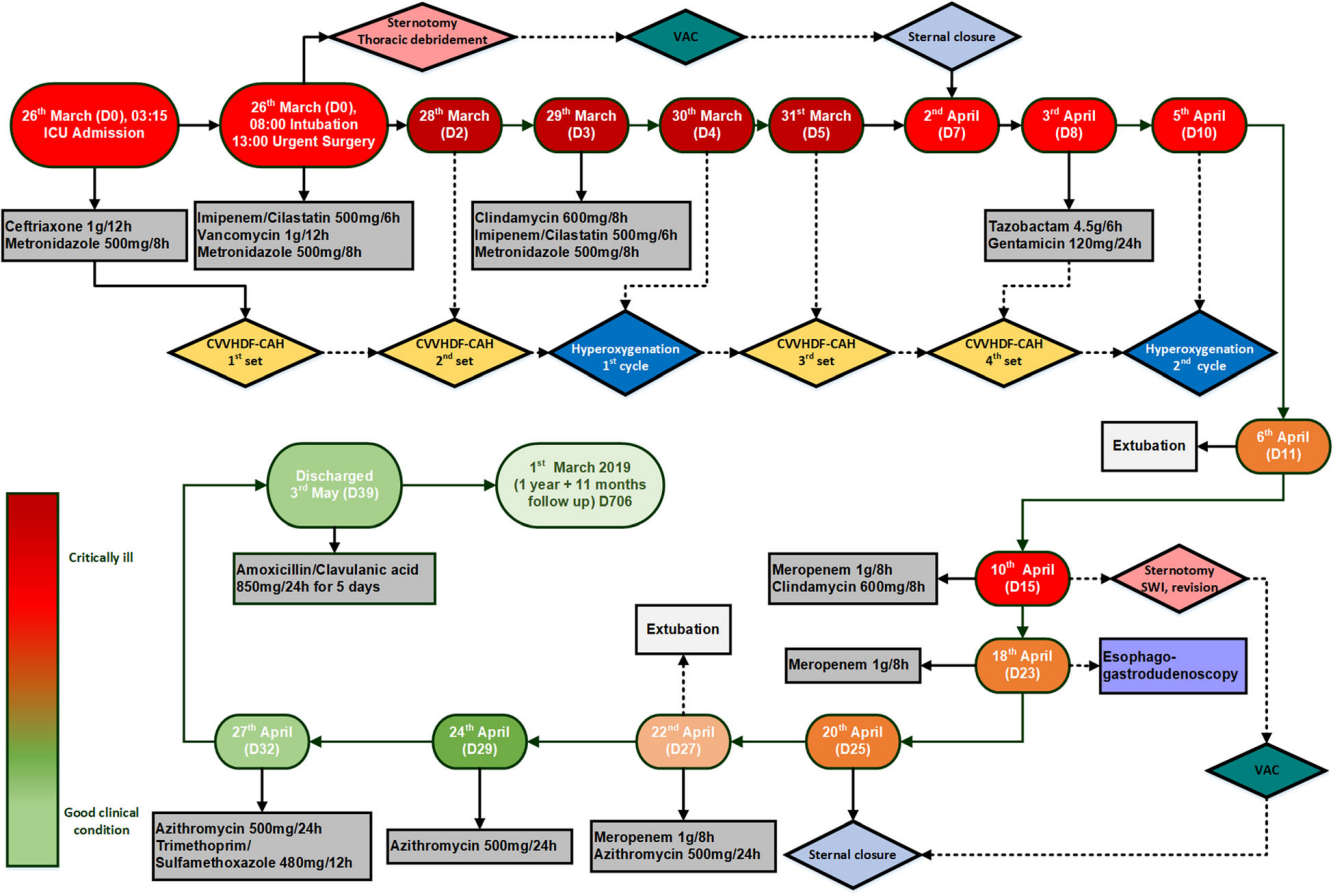
A complete overview of the rotating antibiotic treatment schedule. Flowchart presents detailed antibiotic scheduling and dosages used. The color diagram indicates the health condition of the respective patient

**Fig. 3:**
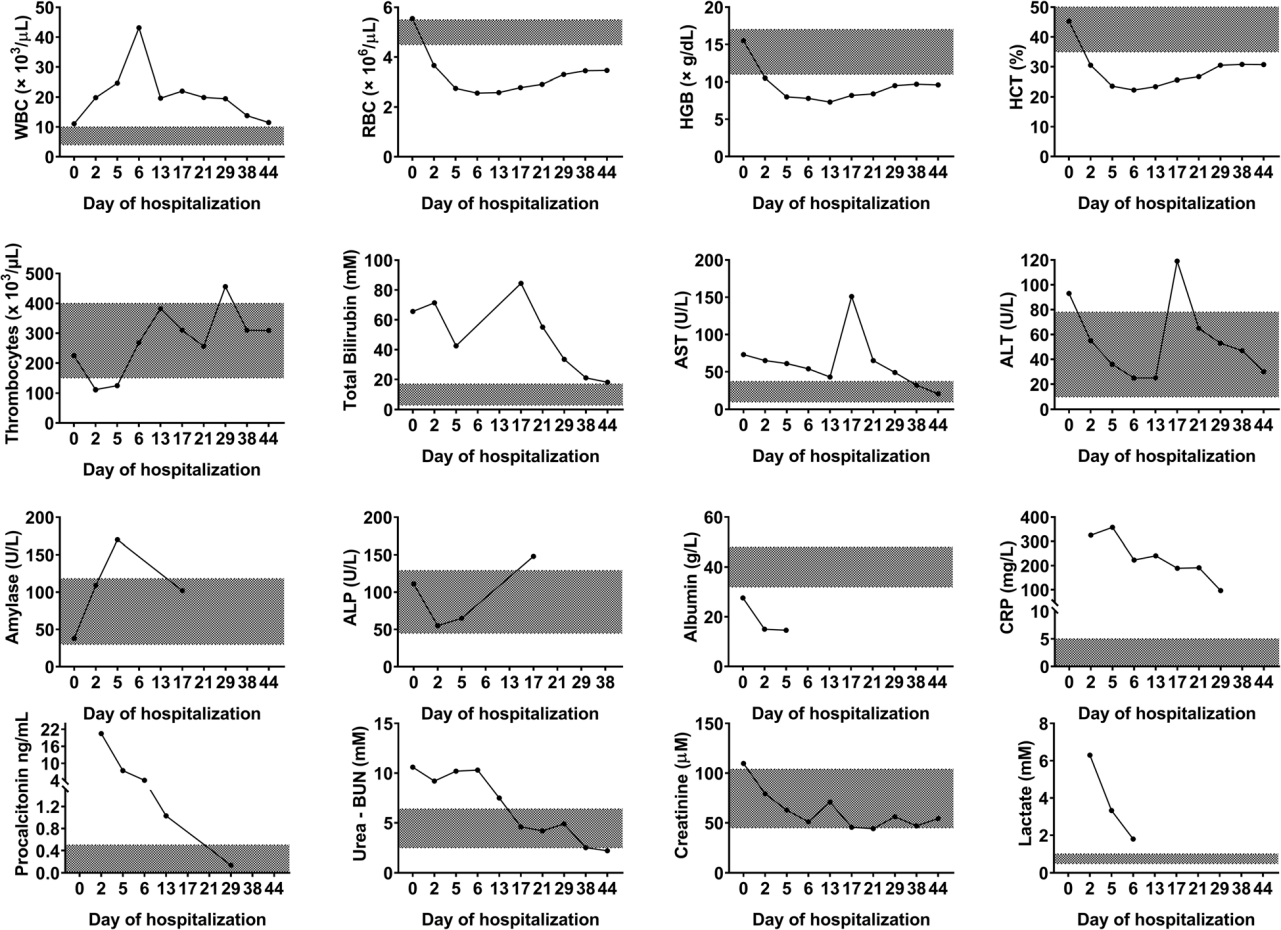
Biochemical parameters measured during hospitalization. Panels display analytical results of various biochemical parameters measured throughout the hospitalization of a critically ill patient with anaerobic septic shock resulting from descending necrotizing mediastinitis. These values were monitored to assess the response to treatment. *ALP* Alkaline phosphatase, *ALT* Alanine aminotransferase, *AST* Aspartate aminotransferase, *BUN* Blood urea nitrogen, *CRP* C-reactive protein, *HCT* Hematocrit, *HGB* Hemoglobin, *RBC* Red blood count, *WBC* White blood count

We collected blood, urine, and tracheal aspirate samples for microbiological analysis (Additional file 1). In anticipation of the definitive culture results, we administered broad-spectrum antibiotics (Fig. 2). The patient was oliguric at this stage, with a urine output less than 50 ml in the first 2 hr in the ICU; furthermore, his creatinine (109.7 μmol/L), urea (10.6 mmol/L), and glomerular filtration rate (GFR) (69 ml/min/1.73 m^2^) pointed to mildly reduced kidney function (Fig. 3).

On the basis of these findings, CRRT was indicated. We placed a 12-French double-lumen catheter in the right femoral vein to establish vascular access. Bearing in mind the likelihood of a systemic infection, we treated the patient with continuous venovenous hemodiafiltration with cytokine-adsorbing filters (CVVHDF-CAH) (PRISMAFLEX oXiris*®* filter; Baxter, Deerfield, IL, USA) set at a high hemofiltration rate of 35 ml/kg/hr. We exchanged the filters every 24 hr.

We next performed an urgent median sternotomy to remove the abscesses and purulent fluid. Tissue samples from the mediastinum and thymic area were collected for microbiological and histopathological analysis. We performed vacuum-assisted wound closure, and we replaced the drain every 48 hr.

The surgical intervention failed to revert his deteriorating condition. He became febrile (38.9 °C) and hemodynamically unstable (Table 1). At this stage, his serum lactate was 6.3 mmol/L, and blood gas analysis (BGA) revealed acidosis. Because of his hemodynamic instability, we administered norepinephrine, but increasing the dosage from 0.2 mg/hr to 1 mg/hr over the next 48 hr was unsuccessful in reverting his poor systemic vascular resistance (FloTrac/Vigileo™ monitoring; Edwards Lifesciences, Irvine, CA, USA) of ≤ 700 dyn/s/cm^5^.

**Table 1:** Hemodynamic monitoring of a patient with descending necrotizing mediastinitis-induced anaerobic septicemia during the first 7 days of hospitalization

Time point of hospitalization	Mean arterial pressure (mmHg)	Heart rate (beats/min)	Cardiac output (L/min)	Cardiac index (L/min/m^2^)	Stroke volume (ml/beat)	Stroke volume variation (%)	Systemic vascular resistance (dyn/s/cm^5^)	Base excess (mmol/L)
Day 0	82	130	ND	ND	ND	ND	ND	ND
Day 1	50	130	4.0	1.8	58	20	≤ 550	−9.1
Day 4	55	120	3.8	1.7	50	23	600	−6.8
Day 6	70	110	4.3	2.0	60	10	800	3.4

*Abbreviation: ND* No data

Extracorporeal blood hyperoxygenation was performed on days 4 and 10

His clinical condition further deteriorated. He developed a high fever (40 °C) overnight, and his Simplified Acute Physiology Score II of 63 points suggested a poor prognosis.

**Fig. 4:**
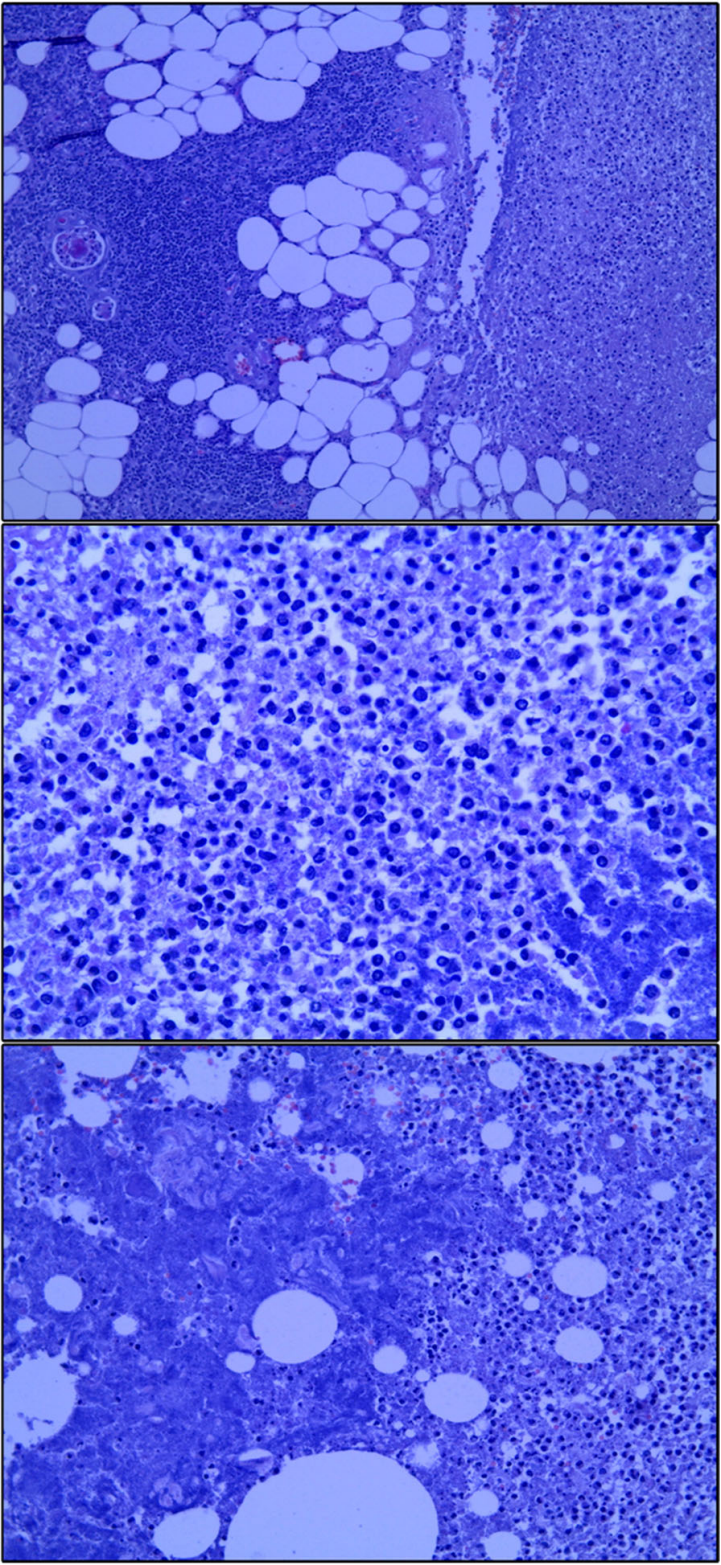
Histopathological staining of the thymic and parathymic tissue. Thymic and parathymic tissue with necrotic debris, neutrophil apoptosis, and massive infiltration of lymphocytes. Hassall’s corpuscles are shown. Lymphocyte aggregates are present in the fat of the involuted thymic tissue. The surrounding connective tissue is characterized by inflammation and purulent (suppurative) exudate consisting of dead neutrophils, fibrinogen, erythrocytes, and colonies of bacteria

Histopathological analysis revealed the presence of acute suppurative inflammation in the thymic and parathymic tissue (Fig. 4), characterized by lymphocytic, neutrophilic infiltration and pus formation. Microbiological analysis of the wound samples revealed gram-positive cocci, identified as α-hemolytic *Streptococcus*; furthermore, the anaerobic cultures of the mediastinal tissue samples confirmed the growth of gram-positive bacilli of the *Clostridium* species.

We switched to a (rotating) antibiotic therapy according to the antibiogram or clinical observations. The detailed antibiotherapy timeline is shown in Fig. 2.

Collectively, the clinical manifestations and radiographic, biochemical (Fig. 3), microbiological, and histopathological examinations indicated a severe case of anaerobic sepsis resulting from DNM [13, 14].

The patient remained in critical condition on day 3, with a fever of 40.1 °C, hemodynamic impairment, acidosis, and lactate level of 3.3 mmol/L (Table 1). We commenced hyperalimentation to counter the patient’s low serum albumin level (14.6 g/L).

On day 4, the patient’s PaO_2_/FiO_2_ ratio was 114 mmHg. A transthoracic echocardiogram revealed pleural effusion in the right costophrenic space. Because of the patient’s worsening condition, we opted for extracorporeal blood oxygenation (EBOO) for 1 hr to improve the peripheral oxygenation of < 90%. EBOO was performed through the (same) right femoral vein catheter used for the CVVHDF-CAH. We coinfused low-dose O_3_, 10 μg/ml blood, and a bolus injection of 2 g of vitamin C to counter possible oxidative stress associated with hyperoxia. We also performed thoracentesis to remove 2800 ml of purulent fluid.

Our approach improved the patient’s clinical condition in the following 24 hr, gradually normalized his body temperature and biochemical parameters, and improved his BGA parameters; his PaO_2_ increased to 433 mmHg from an initial 80 mmHg (Table 1 and Fig. 3).

Consequently, we could decrease FiO_2_ to 50% while maintaining the PaO_2_ at 133.3 mmHg and O_2_ saturation at 98%. The sternum was closed on day 7, and the norepinephrine dosage was lowered to 0.06 mg/hr. The patient was extubated on day 11.

His recovery was short-lived. He once again became febrile on day 15. We observed a purulent secretion from the sternum that required an emergency median sternotomy, surgical debridement of necrotic tissue, and *de novo* vacuum-sealing drainage.

His overall clinical condition improved; nevertheless, he developed gastroesophageal bleeding on day 23. Via esophagogastroduodenoscopy, we identified a polypoid mass on the lateral wall of the esophagus measuring 10–15 mm in diameter. Pathological analysis of the biopsies confirmed squamous papilloma of the esophagus, inflammation, and ulceration.

The bleeding ceased upon the successful excision of the polypoid mass.

From day 24 onward, the patient’s condition steadily improved. He was kept in the ICU on continuous respiratory support and physical therapy until follow-up culture results were negative.

We performed the final vacuum sealing with definitive closure of the lesion on day 25 and extubation on day 27.

**Fig. 5:**
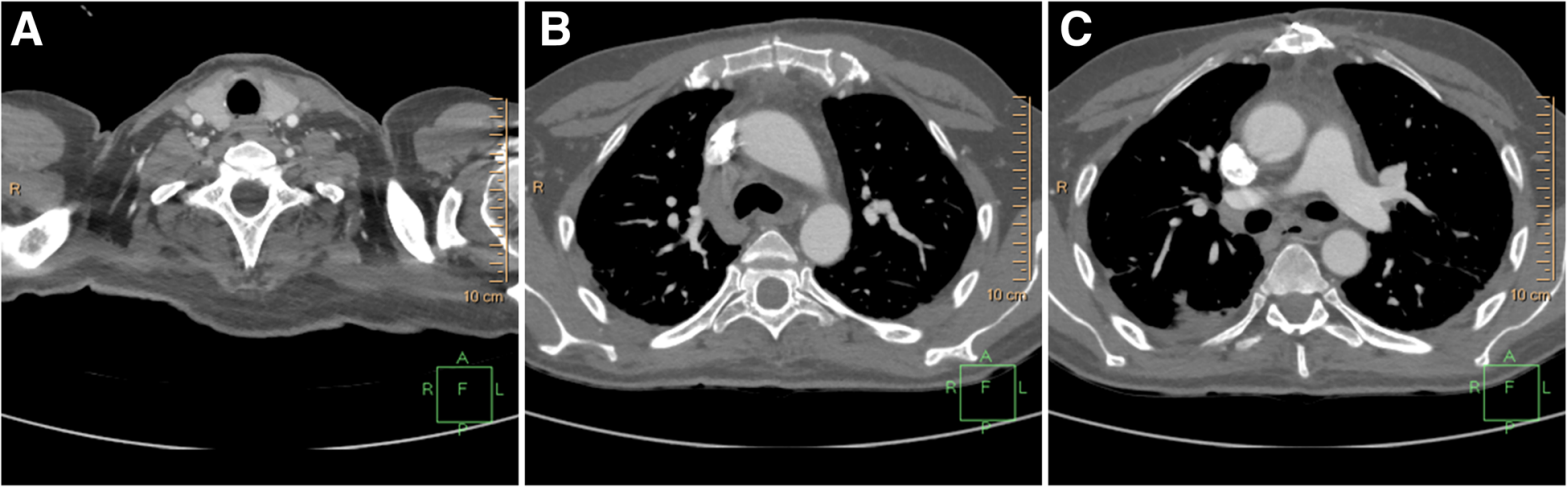
Control computed tomographic analysis. **a** Control computed tomographic scan obtained on day 44 shows normal physiological findings indicating a complete regression of the abscess formation near the left lobe of the thyroid gland and ventral of the carotid arteries. **b** and **c** Fibrous adhesion in the middle mediastinal space

The patient was slightly dysphonic but otherwise in a decent health condition (Fig. 2), and he was discharged on day 39. Control CT evaluation 5 days after discharge confirmed a complete regression of the abscesses (Fig. 5).

The fully recovered patient presented for follow-up 1 year later in good mental and physical condition with no further complications.

## Discussion

In this report, we describe a novel treatment approach for mediastinitis and anaerobic sepsis that includes our early initiation of a CVVHDF-CAH protocol, adjuvant blood purification using the modified AN69ST high-permeability membrane (oXiris®), and the use of intermittent extracorporeal blood hyperoxygenation. Furthermore, our work suggests a clinical benefit of the use of the oXiris® membrane in the treatment of gram-positive bacterial infections, thus possibly paving the way for clinical use beyond gram-negative systemic infections.

Sepsis is characterized by an uncontrolled immune response to infections that may lead to life-threatening organ dysfunction and, in severe cases, death.

Advanced extracorporeal blood purification modalities that include cytokine and endotoxin removal capacity have been developed in the past decade as a potential therapy for critically ill patients with sepsis or those at high risk of developing sepsis and acute kidney injury.

Several extracorporeal blood purification techniques exist [15]. The use of the AN69ST membrane (oXiris®) [16] may offer advantages over other commonly used cytokine-adsorbing hemofilters because it effectively removes a broad spectrum of proinflammatory cytokines [17, 18].

However, the current evidence to support the use of extracorporeal blood purification approaches in humans is limited mostly to case series [19, 20]. Randomized controlled trials have generated conflicting results [21–26]. Consequently, routine clinical use is currently not advocated [27, 28].

Nevertheless, we hypothesize that the heterogeneity in clinical outcomes mirrors the lack of standardized CRRT protocols using blood purification modalities, for instance, the timing of CRRT initiation, CRRT duration, and differences in the type of hemofilter used [29–32].

At our clinic, we consider CVVHDF-CAH (oXiris®) for all hemodynamically unstable (oliguric) critically ill patients who show signs of an uncontrolled inflammatory syndrome (C-reactive protein > 40 mg/ml and procalcitonin > 0.5 ng/ml) in combination with reduced GFR. These include patients suspected of systemic inflammatory response syndrome or sepsis.

With this case report, we present one of our most challenging cases of septic shock resulting from DNM. DNM is a rare but rapidly progressing life-threatening disease in which the mediastinal infection can quickly extend below the tracheal carina, followed by lethal septicemia if not diagnosed and managed in a timely and appropriate manner. In our patient’s case, we suspected the DNM to be a complication of tonsillitis.

Kocher *et al.* and Palma *et al.* recently described their single-center experiences with DNM that signified the complex and heterogeneous clinical manifestation of the disease resulting in varying clinical outcomes [33–37].

Anaerobic infections are challenging in part because of the emergence of antibiotic-resistant strains [12], an unfortunate development especially in the Balkans [38].

The crude mortality associated with anaerobic bacteremia was shown to be 25% [6]. Polymicrobial infection is a known risk factor for death. Progressive infection is often detrimental; uncontrolled anaerobic sepsis carries a high 30-day mortality rate of 51% [3–5].

The European Association for Cardio-Thoracic Surgery recently published its updated recommendations on the management of DNM [1]. In agreement with these recommendations, we directly initiated an aggressive antibiotic treatment plan on suspicion of DNM that was rotated to counter possible antibiotic resistance. Antibiotherapy was followed by a targeted mediastinal surgical debridement of the necrotic tissue, vacuum-assisted wound closure, pleural and pericardial drainage, vasopressor support, and continuous mechanical ventilation.

Nonetheless, the recommended approach was unsuccessful; in these cases, we propose the use of adjuvant therapies in the management of DNM. Our patient presented with DNM and developed acute anaerobic septic shock as a consequence of coinfections by α-hemolytic *Streptococcus* and *Clostridium* species of an unknown source.

*Clostridium* species and α-hemolytic *Streptococcus* induce epithelial cell necrosis, which triggers the release of proinflammatory cytokines and high mobility group protein B1 (HMGB1), a well-described danger-associated molecular pattern associated with sepsis [39, 40]. The AN69ST membrane has a superior adsorptive capacity for endotoxin, lipid A, and, importantly, HMGB1 [16].

Our patient likely benefited from the early use of CVVHDF-CAH (oXiris®) set at a high filtration rate to achieve adequate removal of harmful proinflammatory mediators [19, 41–43]. Also, the use of intermittent extracorporeal blood hyperoxygenation [44] promotes peripheral tissue vascularization [45, 46].

In summary, our combination therapy was successful; the patient recovered completely after a lengthy hospitalization. He regularly communicates with our clinical staff via social media and reports a high quality of life.

## Conclusions

Adjuvant therapy should be considered when first-line treatment regimens fail to control sepsis progression and organ failure.

Early initiation of CVVHDF-CAH may be critical to control systemic infection and provide adequate renal support. Moreover, extracorporeal hyperoxygenation could overturn tissue hypoxia by optimizing peripheral vascularization.

Nevertheless, in the absence of established guidelines advocating the use of CVVHDF-CAH and extracorporeal hyperoxygenation, we conclude that well-designed randomized controlled trials are warranted to guide clinical decision making. Perhaps the ongoing NCT02600312, NCT02398019, and NCT01779635 trials will provide a definitive answer on the clinical benefit of the oXiris® system.

In summary, effective management of DNM-induced anaerobic septic shock benefits from a multidisciplinary approach that builds on the timely administration of antibiotics, skilled surgical intervention, and the use of adjuvant treatment modalities to support renal, pulmonary, and hemodynamic functioning.

## Additional file

Additional file 1: Microbiology screening approach for wound swabs and blood cultures. (DOCX 15 kb)

## Data Availability

Requests for all original data described in this case report can be submitted for evaluation upon request.

## Abbreviations

ALP: Alkaline phosphatase
ALT: Alanine aminotransferase
AST: Aspartate aminotransferase
BGA: Blood gas analysis
BUN: Blood urea nitrogen
CRP: C-reactive protein
CRRT: Continuous renal replacement therapy
CT: Computed tomography
CVVHDF-CAH: Continuous venovenous hemodiafiltration using cytokine-adsorbing hemofilters
DNM: Descending necrotizing mediastinitis
EBOO: Extracorporeal blood oxygenation
GFR: Glomerular filtration rate
HCT: Hematocrit
HGB: Hemoglobin
HMGB1: High mobility group protein B1
ICU: Intensive care unit
PaO_2_/FiO_2_: Ratio of partial pressure of arterial oxygen to fraction of inspired oxygen
RBC: Red blood count
WBC: White blood count
