# Supplementary material for: Continuous venovenous hemodiafiltration using cytokine-adsorbing hemofilters as adjuvant therapy for anaerobic descending necrotizing mediastinitis: a case report
Source: J Med Case Rep. 2019 Jul 5;13:205. doi: 10.1186/s13256-019-2123-7 (PMC6610986; doi:10.1186/s13256-019-2123-7)
Supplement: Supplementary file 1 — Microbiology screening approach for wound swabs and blood cultures. (DOCX 15 kb) [file 13256_2019_2123_MOESM1_ESM.docx]

**Standardised microbiology screening approach for wound swabs** **and blood cultures**

- Cutaneous swabs, sternal swabs and mediastinal swabs were taken and subsequent microbiological determination performed according to our internal JCI compliant SOP. Briefly, samples were collected and transported in agar gel (Swab Stuart, Copan). The samples were cultured on 1) Columbia agar with 5% sheep blood (BioMerieux, France) for 18 hours at 37 ° C with 5% CO2, 2) Schaedler agar (BioMerieux, France) for 48 hours at 37 ° C under anaerobic conditions (GENbox anaer. BioMerieux, France), 3) liquid growth medium, Thioglycolate broth with resazurin (bioMerieux, France) for 48 hours at 37 ° C. and 4) selective media for Staphylococcus (SA Select, BioRad) for 18 hours at 37 ° C, 5) selective media for Gram-negative bacteria (McConkey agar, BioRad) for 18 hours at 37 ° C and 6) direct smear for microscopic examination via Gram-staining.
- Blood samples are inoculated into aerobic and anaerobic culture bottles (BacT/ALERT FN and FA) in the Bact Alert 3D system (BioMerieux, France) and incubated at 37 ° C for 10 days.
- Antimicrobial susceptibility was assessed using EUCAST standards (European Committee on Antimicrobial Susceptibility Testing).
